# Evaluation of the Effect of Virgin Rice Bran Oil (VRBO) on Doxorubicin-induced Cardiotoxicity in Wistar Rats

**DOI:** 10.2174/011573403X327970250108045235

**Published:** 2025-01-28

**Authors:** Suseela Prema, Rakesh Verma, Amritesh Nagarwal, Meenakshi Bharkatiya, Madhuri Baghel, Ladli Kishore, Pranay Wal, Amin Gasmi

**Affiliations:** 1 Crescent School of Pharmacy, BS Abdur Rahman Crescent Institute of Science and Technology, Vandalur, Chennai, 600048, India;; 2 Department of Pharmacology, Institute of Medical Science, BHU, Varanasi, India;; 3 Department of Cardiology, National Institute of Medical Sciences and Research, NIMS University, Jaipur, Rajasthan, 302131, India;; 4 BN Institute of Pharmaceutical Sciences, BN University, Udaipur, India;; 5 Department of Pharmacy, Apollo College of Pharmacy, Anjora, Durg, CG, India;; 6 Department of Pharmacology, Maharaja Agrasen University, Atal Shiksha Kunj, Baddi, Distt., Solan, H.P., India;; 7 Department of Pharmacy, PSIT-Pranveer Singh Institute of Technology Pharmacy, NH 19 Bhaunti Kanpur Agra Highway, Kanpur, UP, India;; 8 Department of Nutrition, Société Francophone de Nutrithérapie et de Nutrigénétique Appliquée, Villeurbanne, France;; 9 International Institute of Nutrition and Micronutrition Sciences, Saint-Étienne, France

**Keywords:** Doxorubicin, cardiotoxicity, virgin rice bran oil, doxorubicin-induced cardiotoxicity, Wistar rats, lactate dehydrogenase, creatinine kinase

## Abstract

**Introduction:**

The usage of doxorubicin (DOX), an antineoplastic drug that is frequently used for the cure of cancer, is restricted to maximal doses due to its cardiac toxicity. Reactive oxygen species produced by DOX result in lipid peroxidation and organ failure, ultimately resulting in cardiomyopathy. Due to its high polyphenol content, virgin rice bran oil (VRBO) is a diet nutritional supplement with a strong antioxidant. This study aimed to assess the potential defense of VRBO against DOX-induced cardiotoxicity.

**Methods:**

VRBO and DOX injections were administered to thirty male Wistar rats for 42 days after being randomly assigned to five groups.

**Results:**

The study demonstrated the cardioprotective effects of VRBO against doxorubicin (DOX)-induced cardiotoxicity. VRBO (0.71 and 1.42 ml/kg) significantly improved the heart-to-body weight ratio, reduced elevated serum CK-MB and LDH levels by 18.4% and 52.7%, respectively, and increased HDL by 43.1%. ECG parameters also improved, with reductions in QT interval (19%), ST interval (28%), and QRS complex (15%). VRBO enhanced systolic blood pressure (up to 21%) and heart rate (7.1%). Antioxidant markers showed notable recovery, with MDA levels reduced by 66.1%, while GSH, SOD, and catalase levels increased by 129.4%, 158.2%, and 84.8%, respectively.

**Conclusion:**

A cardioprotective benefit was found at middle and higher VRBO dosages. In order to demonstrate the effectiveness of VRBO as a cardioprotective medication, further research on dosage response and bioavailability is required.

## 1 INTRODUCTION

Doxorubicin, a quinone-containing anticancer anthracycline antibiotic, has been extensively used to treat various cancers, including breast cancer, liver carcinoma, and bladder cancer. It works well as supplemental and curative chemotherapy, as well as in the alleviation of malignancy symptoms in middle- to late-stage disease [1, 2]. However, common side effects, such as fibrosis and cardiac dysfunction, limit the anticancer administration of DOX. Cardiotoxicity is a major detrimental effect that limits its clinical use. Once symptoms of doxorubicin-induced cardiomyopathy appear, the prognosis is often poor, and the condition is frequently fatal [3-5].

Patients were estimated to develop DOX cardiotoxicity in 5%, 26%, and 48% of cases, respectively, at cumulative doses of up to 400, 550, and 700 mg/kg [6]. It has been discovered that apoptosis, autophagy, calcium dysregulation, DNA damage, oxidative stress, lipid peroxidation, and mitochondrial injury all contribute to the development of DOX-induced cardiotoxicity [2, 7]. Doxorubicin generates semiquinone radicals by losing an electron, which contributes to its cardiotoxic effects. This radical reduces oxygen to produce superoxide, which regenerates doxorubicin. After doxorubicin triggers the reduction of oxygen by NADPH, the antioxidant enzyme superoxide dismutase (SOD) decreases the superoxide radical to hydrogen peroxide (H_2_O_2_) [8]. Fe^2+^ further breaks down H_2_O_2_ into the incredibly reactive hydroxyl radical (OH), which, when combined with polyunsaturated fatty acids, produces lipid hydroperoxide that can produce lipid hydroperoxide through a reaction with polyunsaturated fatty acids. This starts a chain reaction of lipid radicals that can oxidatively degrade cell membranes [9].

Virgin rice bran oil (VRBO) is obtained through a wet extraction process at low temperature from rice bran oil, without the need for refining, bleaching, or deodorizing processes [10]. This process retains the biological activities of the beneficial components in VRBO in contrast to the dry method used to extract rice bran oil [11, 12]. Medium-chain fatty acids make up its primary composition. It acts as a cardioprotective by improving lipid profiles, reducing oxidative stress, and alleviating cardiac remodeling [13]. Moreover, it restores autonomic balance, enhances endothelial nitric oxide synthase expression, and mitigates insulin resistance, offering significant benefits for managing cardiovascular risks associated with metabolic syndrome​ [14].

It has also demonstrated significant cardioprotective effects. In hypertensive models, RBO reduced systolic blood pressure by 21.5%, decreased plasma ACE activity by 59%, and lowered oxidative stress markers like malondialdehyde by 45%. It increased nitric oxide bioavailability by enhancing eNOS expression and reducing inflammatory markers like TNF-α and VCAM-1 expression by over 50%, showcasing its cardiovascular benefits​ [15]. Studies reported that it significantly lowers total cholesterol (7.29 mg/dL), LDL cholesterol (7.62 mg/dL), and triglycerides (9.19 mg/dL), while HDL cholesterol remains unaffected. Its key bioactives, like γ-oryzanol and tocotrienols, regulate cholesterol production and support lipid metabolism [11].

Owing to the presence of tocopherols, γ-oryzanol, and tocotrienols, rice bran oil (RBO) has several advantages over other cooking oils, including oxidative stability and many health benefits [16]. γ-Oryzanol is a class of RBO. Despite being implicated in the pathogenesis of DOX-induced dyslipidemia, oryzanol remains unaffected. Gamma-oryzanol, a compound composed of ferulic acid esters of sterol and triterpene alcohols, is a natural antioxidant found in rice bran oil at a concentration of 1-2% [17]. Recent studies have demonstrated its potential to decrease blood cholesterol levels and decrease the risk of coronary heart disease [18, 19]. Several experimental models have demonstrated that the tocotrienol-rich fraction (TRF) reduced the expression of vascular endothelial growth factor (VEGF), which, in turn, lowered lipid parameters in a dose-dependent manner, with an optimum effect at a dose of 8 mg TRF/kg/day. HMG-CoA reductase activity increased after the atherogenic diet was discontinued but remained significantly lower during TRF treatment. TRF also showed a significant decrease in TBARS and conjugated dienes. Based on these results, taking TRF supplements may be very good for your health because they change many bodily functions, such as the levels of certain lipids that can cause heart disease and the antioxidants that fight it in people with high cholesterol [20].

## 2 MATERIALS AND METHODS

### 2.1 Drugs and Chemicals

We obtained doxorubicin from Sigma-Aldrich Chemical Co. (St. Louis, MO, USA). Rice bran oil was purchased from Sigma-Aldrich Chemical Co. (St. Louis, MO, USA), thiopentone sodium from Akums Pvt. Ltd., urethane from Hi-Media Laboratories Mumbai, formaldehyde solution (35% formalin) from Merck Ltd., heparin from Glenlen Pharma Ltd., and normal saline from Baxter Ltd. Moreover, lactate dehydrogenase (LDH), creatine kinase-myoglobin binding (CK-MB), and high-density lipoprotein (HDL) assay kits were purchased from Sigma Aldrich, India. All of the other chemicals used in this study were of analytical grade.

### 2.2 Experimental Animals

We purchased male Wistar rats (200-250 g) (only male Wistar rats were preferred because oestrogen has inherent cardioprotective qualities because of its antioxidant effects and control of nitric oxide generation, which may skew the results. Female rats also suffer changes in their oestrous cycles, which impact hormone levels, such as progesterone and oestrogen) from Hakravati Industries, Kolkata, for the experiments. The rats were kept in polypropylene cages (47 × 34 × 20 cm) with husk bedding changed daily, under a 12-hour light/dark cycle at about 22°C [21]. They had free access to water and were fed a standard pellet diet (Anant Traders, Lucknow, India) once daily.

The study was approved by the Institutional Animal Ethics Committee, following guidelines from the Committee for Control and Supervision of Experiments on Animals (CPCSEA), India (Approval No. 1273/PO/RE/S/09/CPCS EA).

Rats were randomly placed into treatment groups using a random selection method. The researchers assessing outcomes were blinded to the group assignments of the animals to minimize bias. All procedures followed standard protocols to ensure accuracy and minimize variation. Care was taken to handle the animals gently and minimize stress throughout the study.

The experimental groups received varying doses of VRBO, which were selected based on prior studies to ensure relevance to therapeutic and physiological effects. The doses were chosen to represent sub-therapeutic, therapeutic, and supra-therapeutic levels, allowing the evaluation of dose-dependent effects.

### 2.3 The Experimental Group and the Method of Drug Administration

Thirty Wistar rats in total were randomly split into five groups of six male rats each. We supplied distilled water to the normal control group (group 1) for 42 days. Group 2 (DOX control group) received intraperitoneal injections of doxorubicin (3.75 mg/g) in normal saline on the 14^th^, 21^st^, 28^th^, and 35^th^ days, resulting in a cumulative dose of 15 mg/kg. We administered VRBO (0.42, 0.71, and 1.42 ml/kg) (as an oral liquid) orally to Groups 3, 4, and 5 for 42 days, once daily, along with a DOX injection on days 14^th^, 21^st^, 28^th^, and 35^th^ [22].

### 2.4 Serum Parameters

We took blood from the retro-orbital plexus (ROP) under urethane anesthesia one week following the final intraperitoneal doxorubicin treatment (day 42) in order to analyze biochemical markers. We did not add anticoagulant and stored the serum separately at 4°C until processing. We separated the serum by centrifuging it using an Eppendorf cytocentrifuge (Model No. 580, Eppendorf, Hamburg, Germany), maintaining it at 4°C, and running it at a speed of 7000 rpm for 15 minutes [23]. We measured the serum levels of lactate dehydrogenase (LDH), creatine kinase-myoglobin binding (CK-MB), and high-density lipoproteins (HDL) using a spectrophotometer (UV-visible spectrophotometer, Shimadzu, Japan) and commercially available reagent kits, following the manufacturer's instructions (Sigma Aldrich, India).

### 2.5 ECG

We recorded the ECG one week after the last doxorubicin injection, using an eight-channel power lab (AD Instruments, Australia) with LABCHART-7 Pro software. The anesthetized rats were placed in the supine position on a board, positioning electrodes in a lead-like position. The ECG of the experimental rats was tracked for a minimum of 10 minutes, during which their forms of modifications (QT interval, ST-segment elevation, and QRS complex) were noted [24].

### 2.6 Hemodynamic Parameters

A week subsequent to the final intraperitoneal (i.p.) doxorubicin treatment, we used 1.25 g/kg of urethane to put the mice to sleep. Every animal had its right carotid artery cannulated in order to assess the mean arterial blood pressure (MABP), diastolic blood pressure (DBP), systolic blood pressure (SBP), and heart rate (HR). Heparinized saline was inserted into the cannula, which was then attached to a pressure transducer. Using LABCHART 7 Pro software and eight-channel power laboratories (AD Instruments, Australia), we recorded hemodynamic variables.

We took hemodynamic measurements, put the animals to sleep, and extracted the hearts. Moreover, the amounts of myeloperoxidase, glutathione, and superoxide dismutase were measured after homogenizing a portion of the heart in Tris-HCL buffer. The other section of the heart was placed in a 10% neutral buffered formalin (NBF) solution for histopathology [25].

### 2.7 Antioxidant Parameters

#### 2.7.1 Heart Tissue Preparation for the Estimation of Oxidative Stress Marker

After the experiment concluded, the heart was removed from each animal. Then, it was weighed, dissected, cut into small pieces, placed in a chilled 0.25 M sucrose solution, and blotted on filter paper. After homogenizing the tissue in 10% cold Tris hydrochloride buffer (10 mM, pH 7.4), an Eppendorf 5810-R high-speed chilling centrifuge was used (Remi Equipment Pvt. Ltd., Remi Motors Ltd., Mumbai, India) to centrifuge the material for 15 minutes at 0°C at 7500 rpm. Malondialdehyde (MDA), glutathione (GSH), superoxide dismutase (SOD), and catalase (CAT) were all measured using the clear supernatant [22-26].

#### 2.7.2 Lipid Peroxidation Assay (MDA Content)

We used the lipid peroxidation assay, as described by Slater and Sawyer [27], to determine the level of thiobarbituric acid reactive substances (TBARS). Two milliliters of the homogenized tissue supernatant was collected, and two milliliters of freshly made 10% w/v trichloroacetic acid (TCA) was added to the Eppendorf. For fifteen minutes, the blend was left to rest in an ice bath. Then, the resulting mixture was centrifuged at 2500 rpm for 15 minutes at 0°C after 15 minutes. Afterward, 2.0 ml of freshly made 0.67% w/v thiobarbituric acid (TBA) was combined with a clear supernatant solution. The resultant solution was boiled for ten minutes in a bath of boiling water. The mixture was promptly chilled for five minutes in an ice bath. Using 1, 1, 3, 3-tetraethoxypropane as a standard, we evaluated the absorbance of the generated color at 532 nm using a UV/VIS spectrophotometer (JASCO-V-530, Japan) [24-27].

#### 2.7.3 Estimation of GSH

For the estimation of GSH, 1.0 ml of tissue homogenate (supernatant) and 1 ml of 20% TCA were combined, and the mixture was centrifuged at 2500 rpm for 15 minutes at 0°C. Then, 0.25 ml of supernatant was combined with 2 ml of 5’-dithiobis (2-nitro benzoic acid) (0.6 M) reagent. We used phosphate buffer (pH 8.0) to bring the final amount up to 3 ml. At 412 nm, the produced color was measured against a blank for the reagent. To create a standard curve, various quantities (10-50 µg) of standard glutathione were treated as previously mentioned. The amount of decreased glutathione was expressed as µg of GSH per gram of protein [23, 28].

#### 2.7.4 Estimation of SOD Activity

For the estimation of SOD activity, 1.0 ml of tissue homogenate (supernatant) and 1 ml of 20% TCA were combined, and the mixture was centrifuged at 2500 rpm for 15 minutes at 0°C. Then, 0.25 ml of supernatant was then mixed with 2 ml of 5’-dithiobis (2-nitro benzoic acid) (0.6 M) reagent. We used phosphate buffer (pH 8.0) to bring the final volume up to 3 ml. At 412 nm, the produced color versus a blank for the reagent was measured. To create a standard curve, various quantities (10-50 µg) of SOD were treated as previously mentioned. The results were expressed as units of SOD per gram of protein [22].

#### 2.7.5 Estimation of CAT

Using hydrogen peroxide as the substrate and a method centered around direct detection of H_2_O_2_ breakdown, CAT activity was measured. By tracking the exponential disappearance of H_2_O_2_ at 240 nm, we were able to determine the absorbance and represent the data in units/mg of protein. Each enzyme experiment used an ending amount of 3 ml of the substrate and 20 ml of the supernatant from the homogenate of heart tissue. At 250°C, we ran the experiment and recorded the outcome at 240 nm. A unit of CAT activity was equivalent to one mmol of H_2_O_2_ degraded every minute, and we represented enzyme activity as units per gram of protein [29, 30].

### 2.8 Histopathology

We euthanized the animals, removed their hearts, and placed them in a 10% neutral buffered formalin solution. The organ specimens were dehydrated with xylene for one hour each and alcohol at 70, 90, and 100% strength for two hours each. We carried out infiltration and impregnation by treating the organ specimens twice, each time for one hour, with paraffin wax. Paraffin L-molds were prepared using paraffin wax. The specimens were cut into sections of 3-5 mm thickness and stained with hematoxylin and eosin (H&E). The sections were mounted using diethylene phthalate xylene (D.P.X.). The parameters of histopathological assessment of heart sections were hyperemia, cellular infiltration, and necrosis [31].

### 2.9 Statistical Evaluation

The mean values of the data were expressed along with the standard error of the mean (SEM). Differences among groups were determined using a one-way analysis of variance (ANOVA), followed by the post hoc test Dunnet test. The value of *p* < 0.05 was considered significant. All statistical analyses were performed using Statistical Package for Social Sciences version 16.0 software (SPSS, Inc., Chicago, IL).

## 3 RESULTS

### 3.1 Heart-to-body Weight Ratio

The heart-to-weight of the body ratio was significantly (*p <* 0.001) lower in the doxorubicin control group than in the vehicle control group. In contrast to the doxorubicin control group, ingesting VRBO (0.71 and 1.42 ml/kg) for 42 days resulted in a significant (*p <* 0.01 and *p <* 0.001) rise in the heart-to-body weight ratio; nevertheless, the VRBO (0.42 ml/kg)-treated group did not exhibit any significant effects (Table **1**).

### 3.2 Serum Parameter Evaluation

When doxorubicin control rats were compared with normal control rats, there was a substantial decrease in the level of HDL and a significant increase (*p <* 0.001) in the levels of lactate dehydrogenase (LDH) and creatine kinase-myoglobin binding (CK-MB). In comparison to the doxorubicin control group, giving VRBO (0.71 and 1.42 ml/kg) orally for 42 days decreased LDH and CK-MB levels and increased HDL levels (Table **2**). In contrast to the doxorubicin control group, there was no apparent reduction in LDH or CK-MB levels in the VRBO (0.42 ml/kg) treatment group (Table **2**).

### 3.3 ECG Parameter Evaluation

**Fig. (1):**
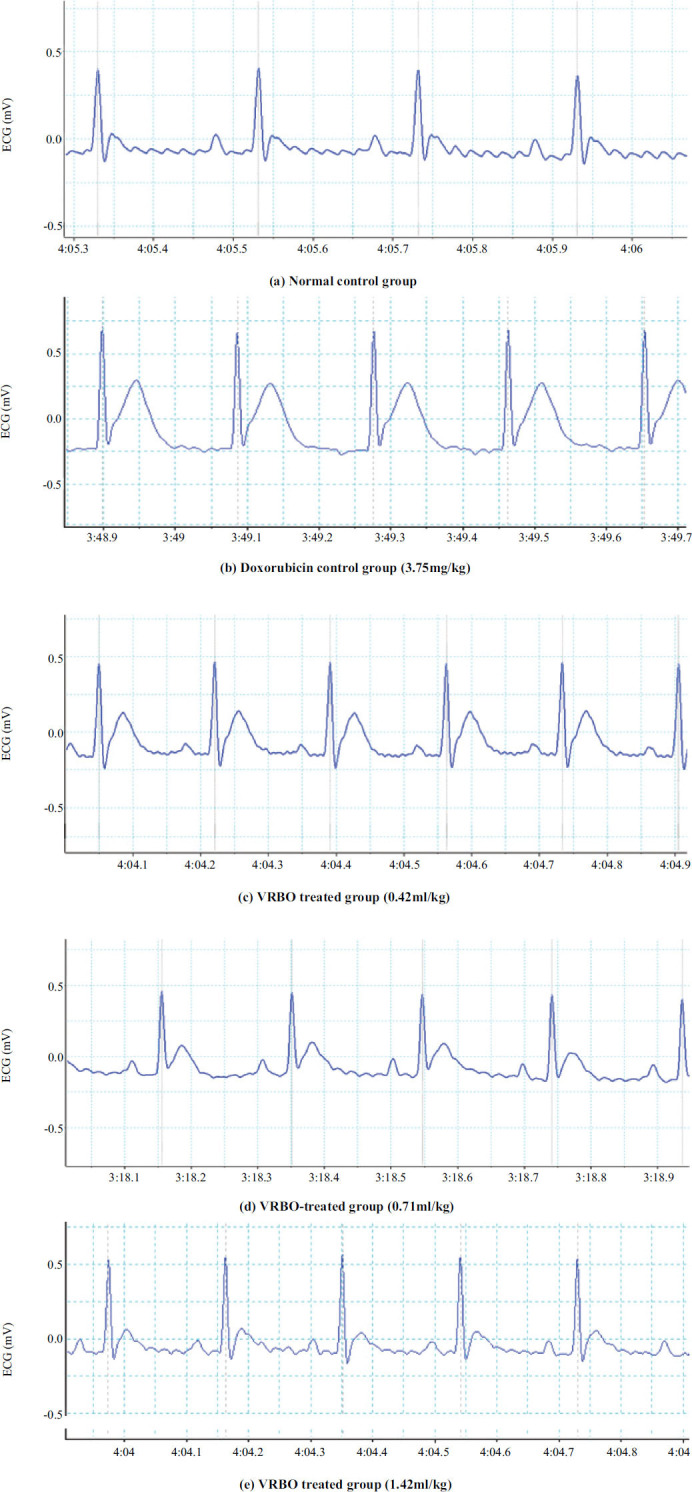
(**a-e**) ECG recording of Wistar rats after administration of doxorubicin and different doses of VRBO.

The QT interval, QRS complex, and ST segment were found to be significantly (*p <* 0.001) higher in the doxorubicin control group than in the normal control group on the 42^nd^ day. The administration of VRBO at 1.42 ml/kg by mouth for 42 days significantly (*p <* 0.001) shortened QT intervals, QRS complexes, and ST segments compared to those who were given doxorubicin. However, VRBO at 0.42 and 0.71 ml/kg did not significantly shorten QT intervals, QRS complexes, or ST segments compared to those who were given doxorubicin (Fig. **1** and Table **3**).

### 3.4 Hemodynamic Parameter Evaluation

The DOX control group exhibited a substantial (*p <* 0.001) drop in systolic blood pressure (SBP), diastolic blood pressure (DBP), and mean arterial blood pressure (MABP) in comparison to the normal control group. When contrast to the doxorubicin control group, the oral administration of VRBO (0.71 ml/kg and 1.42 ml/kg) for 42 days significantly increased the systolic, diastolic, and mean arterial blood pressure (*p <* 0.01 and *p <* 0.001). In contrast to the doxorubicin control group, the VRBO (0.42 ml/kg) treatment group did not exhibit a statistically significant increase. However, in the VRBO-treated group, the rise in blood pressure did not surpass that of the normal control group. On the 42^nd^ day, in the doxorubicin control group, the heart rate (bpm) was lower than that of the vehicle control group. On the other hand, treatment with VRBO at 0.71 ml/kg and 1.42 ml/kg for 42 days showed a significant (*p <* 0.01 and *p <* 0.001) increase in heart rate when compared to the doxorubicin control group; however, treatment with VRBO at 0.42 ml/kg did not show a significant change in heart rate when compared to the doxorubicin control group (Table **4**).

### 3.5 Antioxidant Parameter Evaluation

#### 3.5.1 Malondialdehyde (MDA)

Lipid peroxidation was found to be significantly (*p <* 0.001) increased in the doxorubicin control group when compared with the normal control group. Oral administration of VRBO (0.42, 0.71, and 1.42 ml/kg) for 42 days significantly decreased the MDA level (*p <* 0.01 and *p <* 0.001) (Table **5**).

#### 3.5.2 Glutathione (GSH)

A significant (*p <* 0.001) decrease was found in the glutathione level of the doxorubicin control group when compared with the normal control group. However, treatment with VRBO (0.42 and 1.42 ml/kg) for 42 days significantly increased this level (*p <* 0.01 and *p <* 0.001) (Table **5**).

#### 3.5.3 Superoxide Dismutase (SOD)

SOD (superoxide dismutase) levels were significantly (*p <* 0.001) lower in the DOX control group than in the normal control group. Compared with the DOX control group, administration of VRBO at 0.72 and 1.42 ml/kg significantly and dose-dependently (*p <* 0.01 and *p <* 0.001) increased the level of SOD. However, VRBO at 0.42 ml/kg did not show any significant increase (*p* > 0.05) in SOD levels (Table **5**).

#### 3.5.4 Catalase

Catalase (CAT) levels were significantly (*p <* 0.001) lower in the DOX control group than in the normal control group. Administration of VRBO at 0.71 and 1.42 ml/kg significantly increased the catalase level compared to the DOX control group (*p <* 0.01 and *p <* 0.001), while VCO (0.42 ml/kg) did not show a significant effect when compared to the DOX control group (Table **5**).

### 3.6 Histopathology

Fig. (**2**) shows a section of heart tissue from Wistar rats showing normal histological structure without myocardial degeneration, vacuolation, or inflammation. Administration of doxorubicin resulted in significant histopathological changes. The heart section in Image B displays severe congestion (arrow), myocardial vacuolation (square), and inflammatory cell infiltration. Administration of VRBOO at 0.42 ml/kg resulted in very minimal degenerative changes in the heart myocardium (Image C). Rats pretreated with VRBO at 0.72 ml/kg showed significantly reduced myocardial cell degeneration, and the heart section showed minimal myocardial degenerative changes with minimal congestion. Images D and E present the microscopic and ultrastructural images of VRBO-treated rats (0.72 and 1.42 ml/kg). The administration of VRBO for 42 days markedly reduced myocardial degeneration and inflammation of cells. The VRBO (0.42 ml/kg)-treated group showed mild myocardial degeneration and congestion, whereas the VRBO (1.42 ml/kg)-treated group showed minimal myocardial degenerative changes and minimal congestion.

## 4 DISCUSSION

An effective antineoplastic agent, doxorubicin (Adriamycin), commonly treats various hematological and solid malignancies, including leukemia, lymphomas, osteosarcoma, soft-tissue sarcomas, breast carcinoma, Kaposi's sarcoma, Hodgkin's, and non-Hodgkin's lymphomas [32-36]. Many experimental models have confirmed that late-onset cardiac toxicity, which limits its use, progresses into dilated cardiomyopathy with a high mortality rate [37, 38]. Doxorubicin is converted into its semiquinone form in cardiac tissue, a toxic and short-lived metabolite that reacts with molecular oxygen, generating ROS and ultimately leading to lipid peroxidation [39]. Other possible ways are intercalation into DNA, which stops the production of macromolecules, DNA binding and alkylation, DNA cross-linking, DNA unwinding, DNA strand separation, and helicase activity, and starting apoptosis when topoisomerase II is stopped [10, 40]. An anthracycline-iron (Fe^2+^) free-radical complex is another way by which doxorubicin causes stress [22]. Although dexrazoxone is effective against DOX-induced cardiotoxicity, it enhances DOX myelosuppression, which limits its use [41-43]. However, there is a need for treatments that ameliorate the cardiotoxic effect of doxorubicin.

The primary method for extracting vegetable oils is Soxhlet-based solvent extraction. In this method, the oil seeds were crushed and placed in a packed bed, which was then exposed directly to the solvent, allowing the oil to leach from the solid matrix into the fluid medium. This method yielded yields of 15-20% and 18.4% RBO by weight of rice bran, respectively, using heptane as the solvent and petroleum ether (at 40-60 ^o^C). Liu and colleagues also achieved a yield of roughly 67.73% RBO using Soxhlet extraction by employing hexane. Amongst other nutritious components, RBO often has notably high concentrations of oryzanol, fat-soluble vitamins, sitosterol, and other phytonutrients. Moreover, RBO lowers intestinal cholesterol absorption and boosts cholesterol metabolism, indicating that it could avoid lipid abnormalities and minimize the risk of heart disease [44, 45].

Therefore, we designed the current study to assess how VRBO affects DOX-induced cardiotoxicity. Upon treatment with DOX, there was a significant increase in LDH and CK-MB levels and a decrease in the heart-to-body weight ratio (24-266). Additionally, when the QT interval and ST segment lengthened, the heart rate decreased, which indicated that cardiac damage occurred due to the administration of doxorubicin. These changes were in accordance with observations from previous studies [8, 10, 36].

LDH and CK-MB, which are cardiac biomarker enzymes, demonstrated increased levels of cardiotoxicity [46]. Following myocardial injury, the level of CK-MB and LDH activity in the blood indicated the degree of muscle damage. The amount of LDH and CK-MB significantly decreased in the current investigation in the VRBO-treated groups (0.71 and 1.42 ml/kg), which demonstrated the cardioprotective effect of VRBO at 0.71 and 1.42 ml/kg doses.

Cardiac damage was also evident in the observed ECG changes, such as ST segment prolongation and QT interval. In the doxorubicin control group, there was also a decrease in heart rate, as hypothesized by reactive oxygen species generation, causing disturbances in calcium homeostasis [47]. The excitability of other cells in cardiac cysts and pacemaker cells in the SA node decreased by the drop in intracellular calcium levels. In this research, VCO (0.71 and 1.42 ml/kg) lowered the QT interval and ST segment significantly, indicating that heart damage was stopped. Furthermore, there was a significant change in heart rate upon treatment with VRBO (1.42 ml/kg) when compared to the doxorubicin control group.

Due to its polyphenol content, VRBO is a powerful antioxidant that protects against carcinogens and acts as a scavenger that engulfs the free radicals that cause cell damage by unstable oxygen molecules [35]. Our study demonstrated that using VRBO before DOX led to a significant drop in the levels of MDA and an increase in the levels of antioxidant enzymes like SOD, GSH, and CAT. This indicated that VRBO may help protect the heart. These results are in accordance with those of several other studies, which reported that compounds with antioxidant properties could reduce DOX-induced cardiotoxicity [48]. Famurewa *et al*. (2018) reported that the polyphenol content in VRBO increased the antioxidant status in rats [22].

Compared to the DOX control group, the VRBO-treated group showed fewer cardiotoxic effects at the histopathological level. In our study, we found that the VRBO-treated group had fewer inflammatory cells, less vacuolization, and less cellular infiltration. These results demonstrated the histopathological cardioprotective effect of VRBO against DOX. This study is unique because it is the first to investigate the protective effects of VRBO against doxorubicin-induced cardiotoxicity in Wistar rats. Analysing the data revealed that VRBO provides cardioprotection against damage caused by DOX *via* a number of mechanisms. By reducing lipid peroxidation (lower MDA levels) and strengthening antioxidant defences (higher GSH, SOD, and catalase), it lessens oxidative stress. By raising HDL and lowering cholesterol absorption, VRBO improves lipid profiles, maintains mitochondrial integrity, and stabilises cell membranes. Cellular infiltration and cytokine levels are lower, indicating anti-inflammatory effects. Additionally, it improves haemodynamic stability by enhancing myocardial contractility and vascular function, normalising cardiac electrophysiology, and preventing arrhythmias. These combined effects demonstrate the potential of VRBO as a cardioprotective drug against heart damage caused by chemotherapy.

Although the Wistar rat model provides a dependable method for researching DOX-induced cardiac injury, there are drawbacks to extrapolating these results to humans. The dosages of VRBO utilised may not be directly applicable to human therapies due to differences in rat and human metabolism, drug absorption, and cardiovascular systems. Furthermore, humans and rats may process antioxidants differently. The long-term effects of active ingredients of VRBO, such as tocopherols and γ-oryzanol, are also unknown, including how well they are absorbed and used in human cardiac tissues.

In addition to examining its pharmacokinetics and absorption, future research should concentrate on clinical trials to ascertain the safety, tolerability, and ideal dosage of VRBO in patients receiving DOX chemotherapy. The effects of VRBO on inflammation, calcium homeostasis, apoptosis, and mitochondrial function require mechanistic investigations. Its effectiveness could be further demonstrated by comparison research with other antioxidants and combination treatments using drugs like dexrazoxane. In order to confirm the function of VRBO as a cardioprotective drug in chemotherapy regimens, long-term research should evaluate its effects on delayed-onset cardiotoxicity and total cardiovascular recovery.

## CONCLUSION

The current investigation proved that VRBO protects against the cardiotoxic effects of doxorubicin. According to this study, VRBO may be helpful as a cardioprotective medication, helping chemotherapy patients take doxorubicin more safely. However, with moderate and higher doses of VRBO, we only noticed a cardioprotective benefit. More dose-response and bioavailability research is required to determine the effectiveness of VRBO as a cardioprotective medication. Future research should concentrate on determining the ideal dosage of VRBO to guarantee safety and efficacy in order to implement these findings in clinical practice. To fully understand the potential of VRBO as a cardioprotective medication for chemotherapy patients, more research on how it functions in people or more intricate animal models is also necessary.

## Figures and Tables

**Fig. (2):**
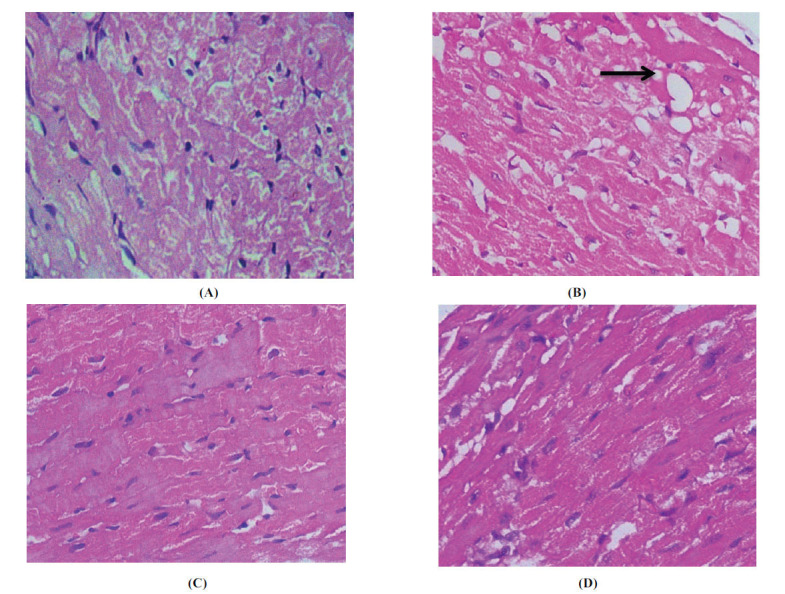
Histopathological changes in rats heart (**A**) Normal control group (normal histological structure without myocardial degeneration, vacuolation, and inflammation) (**B**) Doxorubicin control group (3.75 mg/kg) (severe myocardial degeneration, vacuolation and inflammation) (**C**) VRBO control group (0.42 ml/kg) (**D**) VRBO control group (0.72 ml/kg), (mild myocardial degeneration and congestion); H & E stain, at 100 magnification.

**Table 1:** Effect of VRBO on heart-to-body weight ratio.

**Treatment Group Parameters**	**Normal**	**Doxorubicin**	**Virgin Rice Bran Oil**		
			**0.42 ml/kg**	**0.71 ml/kg**	**1.42 ml/kg**
Heart-to-body weight	3.75 ± 0.12	3.03^###^ ± 0.05	3.31 ± 0.09	3.44 ± 0.07*	3.66 ± 0.1**

**Note: ** Data was expressed as Mean ± SEM, n = 6, one-way ANOVA, followed by Dunnett’s multiple comparison test, ^###^ *p <* 0.001 when compared to normal control, * *p <* 0.05 when compared to DOX control, ** *p <* 0.01 when compared to DOX control.

**Table 2:** Effect of VRBO on serum parameters.

**Treatment Group Parameters**	**Normal**	**Doxorubicin**	**Virgin Rice Bran Oil**		
			**0.42 ml/kg**	**0.71 ml/kg**	**1.42 ml/kg**
CK-MB	757.7 ± 84.9	2532^###^ ± 84.9	2336 ± 83.8	2089** ± 40.7	1399*** ± 109.9
LDH	1687 ± 179.3	5585^###^ ± 192.9	5105 ± 287.8	3527** ± 548.1	2636*** ± 507.5
HDL	47.35 ± 0.92	35.36^###^ ± 1.57	43.53 ± 0.47	47.23*** ± 0.79	50.58*** ± 2.31

**Note: ** Data was expressed as Mean ± SEM, n = 6, one-way ANOVA, followed by Dunnett’s multiple comparison test, ^###^ *p <* 0.001 when compared to normal control, ** *p <* 0.01 when compared to DOX control, *** *p <* 0.001 when compared to DOX control.

**Table 3:** Effect of VRBO on ECG parameter.

**Treatment Group Parameters**	**Normal**	**Doxorubicin**	**Virgin Rice Bran Oil**		
			**0.42 ml/kg**	**0.71 ml/kg**	**1.42 ml/kg**
QT interval (ms)	56.73 ± 1.8	78.71^###^ ± 0.6	72.8 ± 0.58	69.11 ± 2.47	63.88 ± 2.57**
ST interval (ms)	44.79 ± 2.65	61.44^###^ ± 2.13	60.09 ± 1.81	56.11 ± 1.44	44.43 ± 2.08**
QRS complex (ms)	15.02 ± 0.52	17.35^###^ ± 0.68	16.31 ± 0.58	15.42 ± 0.7	14.67 ± 0.51*

**Note: ** Data was expressed as Mean ± SEM, n = 6, one-way ANOVA, followed by Dunnett’s multiple comparison test, ^###^ *p <* 0.001 when compared to normal control, * *p <* 0.05 when compared to DOX control, ** *p <* 0.01 when compared to DOX control.

**Table 4:** Effect of VRBO on hemodynamic parameters.

**Treatment Group Parameters**	**Normal**	**Doxorubicin**	**Virgin Rice Bran Oil**		
			**0.42 ml/kg**	**0.71 ml/kg**	**1.42 ml/kg**
HR (bpm)	365.65±2.29	327.94^###^±1.84	339.37±3.88	343.54**±3.8	351.5**±3.36
SBP (mm/Hg)	125.22±1.52	92.3^###^±0.81	95.41±1.35	102.4*±2.26	112.3**±3.72
DBP (mm/Hg)	81.89±0.9	67.21^###^±0.67	69.34±0.25	75.26***±0.32	86.11***±0.88
MABP (mm/Hg)	104.63±1.33	76.93^###^±1.33	78.89±0.77	88.22**±1.85	103.46***±1.77

**Note: ** Data was expressed as Mean ± SEM, n = 6, one-way, ANOVA followed by Dunnett’s multiple comparison test, ^###^ *p <* 0.001 when compared to normal control, * *p <* 0.05 when compared to DOX control, ** *p <* 0.01 when compared to DOX control, *** *p <* 0.001 when compared to DOX control.

**Table 5:** Effect of VRBO on antioxidant parameters.

**Treatment Group Parameters**	**Normal**	**Doxorubicin**	**Virgin Rice Bran Oil**		
			**0.42 ml/kg**	**0.71 ml/kg**	**1.42 ml/kg**
MDA (nmol/g of protein)	36.65±1.14	127.12^###^±7.97	110.22*±4.63	78.4***±1.6	43.12***±2.04
GSH (µmol/g of protein)	4.97±0.16	1.97^###^±0.15	2.09±0.21	3.21**±0.18	4.51***±0.3
SOD (U/mg of protein)	33.54±0.82	14.45^###^±0.31	16.38±0.5	23.05***±1.46	37.33***±2.49
Catalase (U/mg of protein)	7.78±0.07	3.68^###^±0.32	4.79±0.39	5.73**±0.32	6.8***±0.36

**Note: ** Data was expressed as Mean ± SEM, n = 6, one-way ANOVA, followed by Dunnett’s multiple comparison test, ^###^ *p <* 0.001 when compared to normal control, * *p <* 0.05 when compared to DOX control, ** *p <* 0.01 when compared to DOX control, *** *p <* 0.001 when compared to DOX control.

## Data Availability

The data supporting the finding of the study are available within the article.

## LIST OF ABBREVIATIONS

CAT: Catalase
CCSEA: Committee for Control and Supervision of Experiments on Animals
CK-MB: Creatine Kinase-Myoglobin Binding
DBP: Diastolic Blood Pressure
DOX: Doxorubicin
EDTA: Ethylenediaminetetraacetic Acid
GSH: Glutathione
HDL: High-density Lipoprotein
HMG-CoA: Hydroxymethylglutaryl-CoA
HR: Heart Rate
IAEC: Institutional Animal Ethics Committee
LDH: Lactate Dehydrogenase
MABP: Mean Arterial Blood Pressure
MDA: Malondialdehyde
NBF: Neutral Buffered Formalin
RBO: Rice Bran Oil
ROP: Retro Orbital Plexus
ROS: Reactive Oxygen Species
SBP: Systolic Blood Pressure
SOD: Superoxide dismutase
TBARS: Thio Barbituric Acid Reactive Substances
VEGF: Vascular Endothelial Growth Factor
VRBO: Virgin Rice Bran Oil
